# Usefulness of Intestinal Fatty Acid-Binding Protein in Predicting Strangulated Small Bowel Obstruction

**DOI:** 10.1371/journal.pone.0099915

**Published:** 2014-06-13

**Authors:** Hirotada Kittaka, Hiroshi Akimoto, Hitoshi Takeshita, Hiroyuki Funaoka, Hiroshi Hazui, Masao Okamoto, Hitoshi Kobata, Yasuo Ohishi

**Affiliations:** 1 Department of Emergency, Osaka Mishima Emergency Critical Care Center, Takatsuki, Osaka, Japan; 2 Department of Inspection, Osaka Mishima Emergency Critical Care Center, Takatsuki, Osaka, Japan; 3 DS Pharma Biomedical CO., Ltd, Suita, Osaka, Japan; Gentofte University Hospital, Denmark

## Abstract

**Background:**

The level of intestinal fatty acid-binding protein (I-FABP) is considered to be useful diagnostic markers of small bowel ischemia. The purpose of this retrospective study was to investigate whether the serum I-FABP level is a predictive marker of strangulation in patients with small bowel obstruction (SBO).

**Methods:**

A total of 37 patients diagnosed with SBO were included in this study. The serum I-FABP levels were retrospectively compared between the patients with strangulation and those with simple obstruction, and cut-off values for the diagnosis of strangulation were calculated using a receiver operating characteristic curve. In addition, the sensitivity, specificity, positive predictive value (PPV) and negative predictive value (NPV) were calculated.

**Results:**

Twenty-one patients were diagnosed with strangulated SBO. The serum I-FABP levels were significantly higher in the patients with strangulation compared with those observed in the patients with simple obstruction (18.5 vs. 1.6 ng/ml p<0.001). Using a cut-off value of 6.5 ng/ml, the sensitivity, specificity, PPV and NPV were 71.4%, 93.8%, 93.8% and 71.4%, respectively. An I-FABP level greater than 6.5 ng/ml was found to be the only independent significant factor for a higher likelihood of strangulated SBO (P =  0.02; odds ratio: 19.826; 95% confidence interval: 2.1560 – 488.300).

**Conclusions:**

The I-FABP level is a useful marker for discriminating between strangulated SBO and simple SBO in patients with SBO.

## Introduction

Small bowel obstruction (SBO) is commonly encountered in the field of gastroenterology, the leading cause of which is adhesion in patients with a history of abdominal surgery, accounting for over 70% of cases of SBO [1], [2]. Among the types of SBO, the rate of strangulated SBO has been reported to be lower than that of simple SBO (10% vs. 90%), while the mortality rate is 15–30%, which is much higher than that of simple SBO (5.8–8%) [2]–[4]. Therefore, although it remains challenging, making an accurate diagnosis, in particular discriminating between strangulated and simple SBO, is very important in order to reduce mortality.

In a past report, strangulated SBO was diagnosed based on the presence of one or more classical clinical signs, such as continuous abdominal pain, fever, tachycardia, symptoms of peritoneal irritation, leukocytosis and metabolic acidosis [3]. However, retrospective and prospective studies have found that these individual parameters are not always reliable for detecting strangulated SBO [3], [5]–[9]. Regarding advances in imaging technology, enhanced computed tomography (CT) has recently been introduced as a useful tool for differentiating between simple and strangulated SBO with a high rate of accuracy (73–93%) [10]–[13]. Reduced enhancement of the bowel wall, a serrated beak, thickening of the bowel wall, mesenteric engorgement and ascites have been reported to be useful CT findings enabling the detection of strangulated SBO, with a sensitivity of 33–48%, 32–47%, 38–52%, 19–58% and 64–75% and a specificity of 100%, 100%, 59–95% and 38–86%, respectively [11]–[14]. Nevertheless, detecting these findings remains difficult, even for physicians who specialize in digestive surgery or radiology. The sensitivity and specificity of the diagnosis of intestinal ischemia by experienced gastrointestinal radiologists blinded to patient identification and all clinical information have been reported to be 15–30% and 91–94%, respectively [14]. Therefore, the identification of accurate and simple diagnostic markers that can be used to easily distinguish between strangulated and simple SBO, even by physicians without a specialty in gastroenterology or radiology, is required.

Accordingly, intestinal fatty acid-binding protein (I-FABP), cytosolic proteins with a molecular mass of 14–15 kDa, are expected to be useful in this setting [15]. I-FABP are abundant in the mucosa of the small intestine from the duodenum to the distal segment of the ileum, representing 2.5% of cytosolic proteins in enterocytes situated on villi [15], [16]. Pelsers et al. reported that I-FABP is present in both the small intestine and large bowel, although the I-FABP content in the duodenum, jejunum and ileum is significantly higher than that observed in the colon, and the jejunum in particular contains about 20 times as much I-FABP as the colon[16]. Based on their characteristics, I-FABP are rapidly released into the circulation when the integrity of the enterocyte membrane is compromised, which makes them a potentially suitable biomedical predictor of small bowel ischemia. Several experimental studies investigating the correlation between intestinal ischemia and the serum I-FABP level have reported that the I-FABP levels are significantly increased in intestinal ischemic models compared with that observed in control models [17], [18]. Clinical studies comparing the I-FABP levels between patients with and without intestinal ischemic disease have also shown the same results. Consequently, the I-FABP level is considered to be a useful diagnostic marker of small bowel ischemia [19]–[22]. However, only one study, which included only three patients with necrotic bowel due to strangulation, has reported the usefulness of measuring the I-FABP level in order to distinguish strangulated SBO from simple SBO [20]. Therefore, investigations with a larger number of patients with bowel necrosis due to strangulated SBO are needed. The aim of this retrospective study, which included more patients than previous reports, was to investigate whether the serum I-FABP level is indeed a predictive marker of strangulated SBO by comparing the I-FABP levels between patients with strangulated versus simple SBO.

## Patients and Methods

### Patients

Among a total of 281 patients who were emergently transported with acute abdomen to Osaka Mishima Emergency Critical Care Center between April 2008 and June 2013, those with clinical symptoms and physical findings such as strong abdominal pain, frequent vomiting, a lack of flatus or defecation, abdominal distension, attenuated bowel sounds, and so on, were suspected of having bowel obstruction. Enhanced CT scans were performed in these patients, and those diagnosed with large bowel obstruction were excluded. The remaining patients diagnosed with SBO were included in this retrospective study. The subjects were finally diagnosed with either strangulated SBO or simple SBO based on enhanced CT findings, which were obtained in all patients. Patients with the following CT findings were preoperatively diagnosed with strangulated SBO: a decreased enhancement of the small bowel wall, a closed loop sign with mesenteric vessels converging toward to the site of obstruction, the appearance of ascites and a whirl sign [23]. Those diagnosed with strangulated bowel obstruction were emergently transported to the operating room for immediate surgery, including resection of the necrotic intestine, if necessary, at which time, strangulation was confirmed. Those without CT findings suspicious of strangulated SBO were diagnosed with simple SBO, and first quickly treated with or without the insertion of a long tube. However, surgical procedures, such as adhesiotomy, were required if conservative therapy was ineffective. Patients with strangulated SBO were excluded from the present study if they were considered to be incapable of rescue due to hemodynamic and/or breathing instability. Additionally, patients with chronic kidney disease were excluded because a poor renal function influences the clearance of serum I-FABP. Clinical information, including age, gender, period from onset of symptoms to admission, intraoperative findings, length of stay and laboratory markers, such as the white blood cell (WBC) count, platelet (Plt) count and levels of alkaline phosphatase (ALP), C-reactive protein (CRP), creatine phosphokinase (CK), lactate dehydrogenase (LDH) and lactic acid (LA), were recorded, and the type of bowel obstruction, i.e., strangulated or simple, was ultimately confirmed at discharge taking into consideration the clinical course. This study was approved by the institutional review board, and written informed consent was obtained from all patients.

### Measurement of the serum I-FABP levels

Blood samples for measurement of the serum I-FABP levels were obtained from all 37 patients within 15 minutes after arrival to our institution. The sera were separated from the blood samples and frozen at −20°C in our institution until being transported to D.S. Pharma Biomedical CO., Ltd. (Osaka, Japan) for measurement of the I-FABP levels. The serum I-FABP levels were measured using a sandwich enzyme-linked immunosorbent assay (ELISA) system with rabbit anti-human I-FABP polyclonal antibodies in the solid phase and mouse anti-human I-FABP monoclonal antibodies in the liquid phase, which was described in detail by Funaoka et al. [24]. Using this method, the intra-asssay and inter-assay coefficients of variation (CV) were <2%, and <5%, respectively. I-FABP values ranging from 0.1 to 50 ng/ml can be quantified; thus, blood samples exhibiting a value of 50 ng/ml or more were measured after dilution, and the reference value in the healthy population was defined as below 2.0 ng/ml. The results of the serum I-FABP measurements were reported several months after the blood samples were collected and therefore had no influence on decision making with respect to the treatment strategy for each patient.

### Statistical analysis

Cross tabulations were performed using either Pearson's chi-square test or Fisher's exact test to compare the clinical characteristics between the patients with strangulated SBO and those with simple SBO, where appropriate. Comparisons of continuous variables between the groups were made using the Mann-Whitney U test. P values of <0.05 were considered to be statistically significant. Moreover, for each laboratory marker, a receiver operating characteristic (ROC) analysis was performed, and the area under the ROC curve was acquired. In addition, for each marker, the concentration at which the Youden index [sensitivity – (1 – (specificity)] exhibited a maximum value was selected as the cut-off level for discriminating between strangulated and simple SBO, and the sensitivity, specificity, positive predictive value (PPV) and negative predictive value (NPV) were calculated using the cut-off value. All analyses were performed using the JMP 10 statistical software package (SAS Institute Inc., Cary, NC, USA). The present study adhered to the STAndards for Reporting of Diagnostic Accuracy (STARD) criteria for studies reporting the diagnostic accuracy [25].

## Results

### Patient characteristics

**Figure 1:**
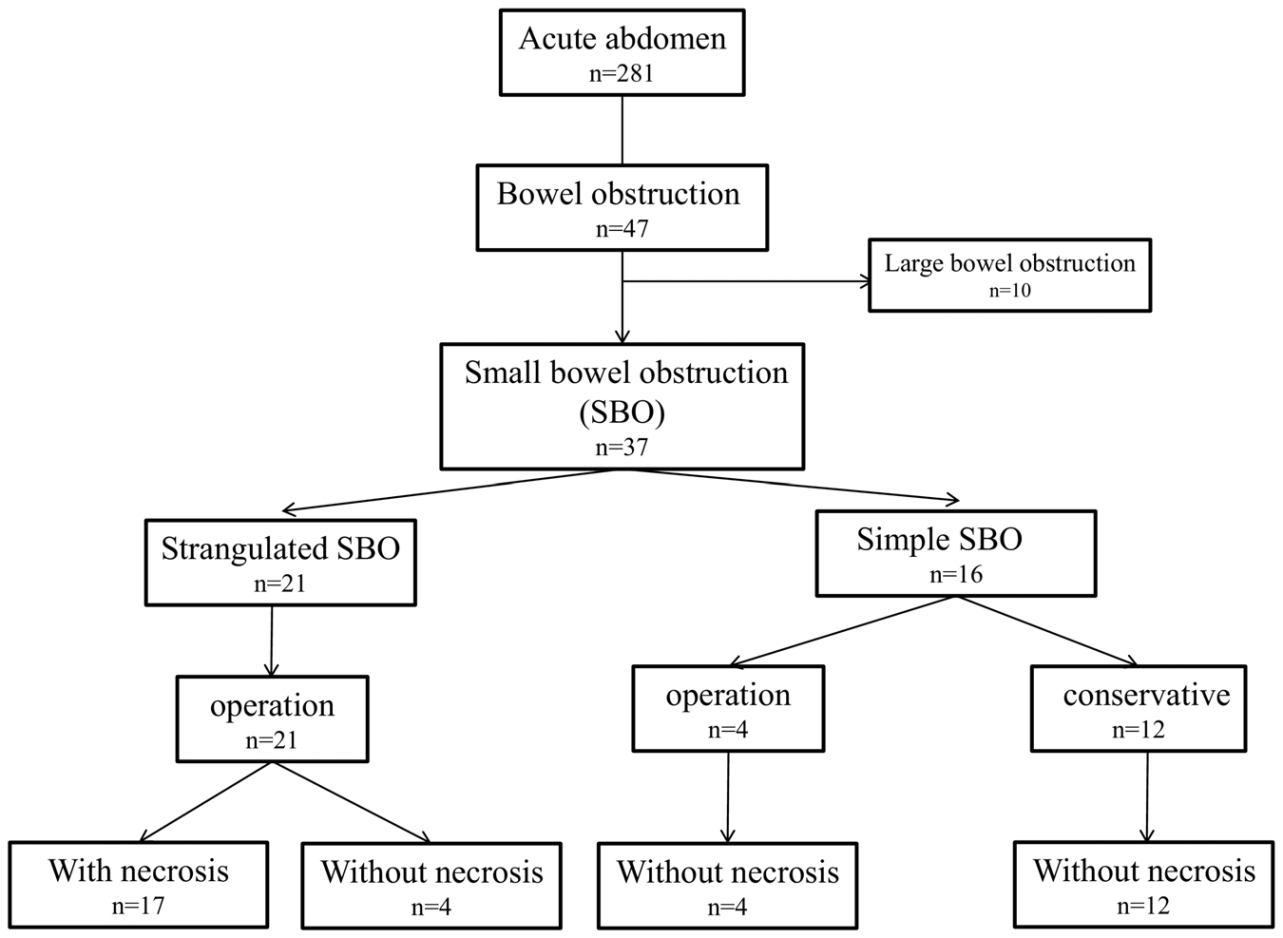
Flow diagram of the patients included in this study. Forty-seven of 281 patients with acute abdomen were diagnosed with bowel obstruction. Ten patients with large bowel obstruction were excluded, and finally 37 patients with small bowel obstruction (SBO) were included in this study. According to the findings of enhanced computed tomography, they were divided into the two groups, those with strangulated bowel obstruction or those with simple bowel obstruction.

Forty-seven patients were suspected of having bowel obstruction, 10 of whom were excluded from the study because they were diagnosed with large bowel obstruction (i.e., four patients were diagnosed with bowel obstruction due to cancer of the colon, while the remaining six patients were diagnosed with torsion of the mesentery of the sigmoid colon). Finally, the remaining 37 patients were included in this study, 21 of whom were diagnosed with strangulated SBO and 16 of whom were diagnosed with simple SBO based on their CT findings (Figure 1). As shown in Table 1, there were 17 males and 20 females, with a median age of 79 years (range: 32–94). Comparing the clinical conditions of the patients on admission between the groups, the systolic blood pressure and body temperature values were significantly lower in the patients with strangulated SBO than in those with simple SBO (108 vs.140 mmHg p<0.001, 36.3 vs. 37.1°C p = 0.02, respectively). The duration from the onset of symptom to admission was not significantly different between the groups (18 vs. 16 hours), and enhanced CT was performed in all patients. The appearance rate of CT findings which were suspicious of strangulated SBO or intestinal necrosis was also shown in Table 1. Compared among the two groups, appearance of ascites, decreased enhancement of intestinal wall, mesenteric edema, and closed loop sign were seen more frequently in patients with strangulated SBO than in those with simple SBO, although the presence of intramural gas and whirl sign did not demonstrate a significant difference. Emergency laparotomy was performed in all patients with strangulated SBO, and an elective operation was performed in four patients (25%) with simple SBO during the time of hospitalization. Seventeen of the 21 patients (81%) with strangulation required resection of the small intestine due to necrosis, while the remaining four patients exhibited resolution of strangulation only because the small intestine fell into an ischemic, but not necrotic, state, although, during the preoperative period, all four patients were suspected of having intestinal necrosis based on the CT findings. Among the 17 patients who required intestinal resection, the length of the necrotic bowel ranged from 50 cm to 180 cm, with a median of 100 cm. The causes of strangulation included the formation of adhesive bands in 11 of 21 patients (52%) and torsion of the mesentery in 10 patients (48%). Among the 16 patients without strangulation, four (25%) required laparotomy because their condition did not improve with conservative therapy (Figure 1). Although enlargement of the oral bowel from the site of obstruction was recognized in all four patients, no patients developed intestinal ischemia. The cause of obstruction was diagnosed according to the operative findings as postoperative adhesion in three patients and an intestinal tumor in one patient. The cause of obstruction was diagnosed in the 12 patients who were successfully treated with non-operative management for simple SBO based on their clinical symptoms, past history, results of blood examinations and CT scans, and clinical courses. Nine of the 12 patients had a past history of laparotomy, and, in all cases, the cause of obstruction was diagnosed to be postoperative adhesion, while the remaining three were diagnosed with paralytic ileus caused by acute enteritis in two patients and due to the side effects of antipsychotic agents in one patient. The length of hospital stay was significantly shorter in the strangulated SBO group than in the simple SBO group (p = 0.02). One patient with strangulation died due to multiorgan failure following the development of leakage at the anastomotic site; no patients with simple SBO died.

**Table 1:** Clinical characteristics (n = 37).

		Total	Strangulated SBO^b^	Simple SBO^b^	p-value
		(n = 37)	(n = 21)	(n = 16)	
Age		79	80	66	0.28
Gender (M,%)		46	33	63	0.10
History of laparotomy (%)		68	71	63	0.57
Systolic blood pressure (mmHg)		120	108	140	<0.001
Heart rate (bpm)		92	98	90	0.26
Body temperature (°C)		36.6	36.3	37.1	0.02
Period from onset to admission (hr)		18	18	16	1.00
CT^a^ scan (%)		100	100	100	1.00
CT findings (%)					
ascites		73	95	44	<0.001
decreased enhancement of the intestinal wall		46	81	0	<0.001
intramural gas		27	33	19	0.33
mesenteric edema		49	76	13	<0.001
closed loop sign		43	76	0	<0.001
whirl sign		11	19	0	0.12
Operation (%)		68	100	25	<0.001
Necrosis of the small bowel (%)		46	81	0	<0.001
Causes of obstruction (n)					
adhesive bands			11		
torsion of the mesentery			10		
simple adhesion				12	
acute enteritis				2	
small intestinal tumor				1	
side effect of drugs				1	
Length of days (n)		11	6	11	0.02
Death (n)		1	1	0	1.00

a: CT indicates computed tomography

b: SBO indicates small bowel obstruction

### Comparison of laboratory markers, with cut-off values for discriminating between cases of strangulated and simple bowel obstruction

**Figure 2:**
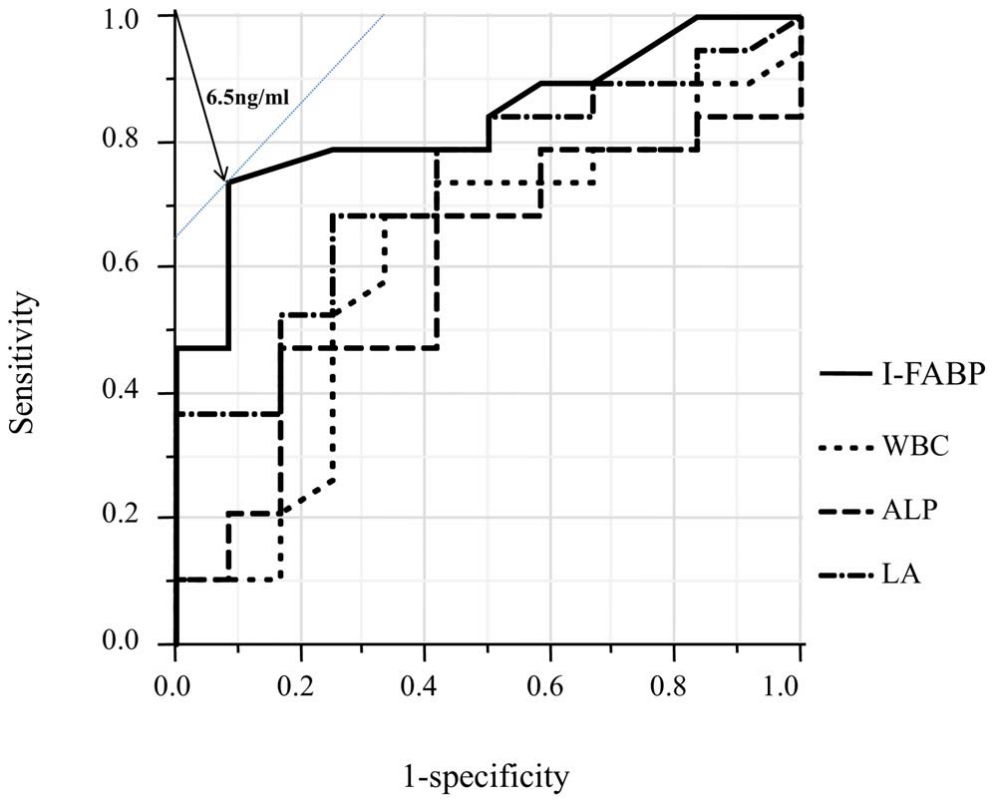
Receiver operating characteristics curves showing diagnostic performance for strangulated small bowel obstruction. Areas under the curve (95% confidence intervals) for each marker are as follows: intestinal fatty acid-binding protein (I-FABP), 0.854 (0.685- 0.941); lactic acid, 0.735 (0.524– 0.894); white blood cell (WBC) count, 0.664 (0.443–0.805); alkaline phosphatase (ALP), 0.640 (0.442–0.799). The cut-off level of I-FABP concentration at which the Youden index exhibited a maximum value was 6.5 ng/ml.

Table 2 shows the median values for each blood marker in the two groups. The I-FABP and LA levels were significantly different between the two groups (18.5 vs. 1.6 ng/ml p<0.001, 3.09 vs. 1.02 mmol/l p = 0.03, respectively).For all markers, a ROC analysis was performed to determine the cut-off value for discriminating between strangulated and simple SBO. Figure 2 shows the ROC curve for each marker with a P-value of less than 0.20 according to a univariate analysis of the two groups. The area under the ROC curve for the I-FABP level was largest among the markers, and the cut-off value for each marker was calculated as described in Table 3. Using these cut-off values, the sensitivity, specificity, PPV and NPV for the I-FABP level were 71.4%, 93.8%, 93.8% and 71.4%, respectively (Table 3). As shown in Table 4, the levels of WBC, LA and I-FABP exhibited significant values for differentiation according to a univariate analysis of the cut-off values.

**Table 2:** Results of the blood examinations.

		Strangulated SBO^a^	Simple SBO^a^	P value
WBC	(/μl)	14100	9850	0.14
Plt	(×10^4^/μl)	21.5	25.0	0.56
ALP	(U/l)	196	228	0.15
LDH	(U/l)	177	172	0.31
CK	(U/l)	99	113	0.94
CRP	(mg/dl)	1.41	1.01	0.91
LA	(mmol/l)	3.09	1.62	0.03
I-FABP	(ng/ml)	18.5	1.6	<0.001

a: SBO indicates small bowel obstruction

WBC: white blood cell; Plt: platelet; ALP: alkaline phosphatase; LDH: lactic dehydrogenase; CK: creatine phosphokinase; CRP: C-reactive protein; LA: lactic acid; I-FABP: intestinal fatty acid-binding protein.

**Table 3:** Comparison of the diagnostic usefulness of the blood biochemical markers for predicting strangulated small bowel obstruction.

	Areas of under the ROC^a^ curve	cut-off value	sensitivity	specificity	PPV^b^	NPV^c^
WBC	0.664	13700	57.1	81.2	80.0	59.1
	(0.443–0.805)					
Plt	0.557	27.8	23.8	56.3	41.7	36.0
	(0.353–0.743)					
ALP	0.640	205	38.1	31.2	42.1	27.8
	(0.442–0.799)					
LDH	0.598	147	85.7	37.5	64.3	66.7
	(0.404–0.766)					
CK	0.493	47	85.7	31.2	62.1	62.5
	(0.303–0.685)					
CRP	0.510	23.2	14.3	100	100	47.1
	(0.325–0.693)					
LA	0.735	2.2	68.4	75.0	81.3	60.0
	(0.524–0.874)					
I-FABP	0.854	6.5	71.4	93.8	93.8	71.4
	(0.685–0.941)					

Numbers in parentheses represent 95% confidence intervals.

a ROC indicates receivor operating characteristics.

b PPV indicates the positive predictive value.

c NPV indicates the negative predictive value.

WBC: white blood cell; Plt: platelet; ALP: alkaline phosphatase; LDH: lactic dehydrogenase;

CK:creatine phosphokinase; CRP: C-reactive protein; LA:lactic acid; I-FABP: intestinal fatty acid-binding protein

**Table 4:** Univariate analysis of biomedical markers predicting strangulated small bowel obstruction.

		Strangulated SBO	Simple SBO	P value
WBC	≧13700	57%	19%	0.02
	<13700	43%	81%	
Plt	≧27.8	24%	44%	0.20
	<27.8	76%	56%	
ALP	≧205	38%	69%	0.06
	<205	62%	31%	
LDH	≧147	86%	63%	0.10
	<147	14%	37%	
CK	≧47	86%	69%	0.21
	<47	14%	31%	
CRP	≧23.2	14%	0%	0.24
	<23.2	86%	100%	
LA	≧2.2	68%	25%	0.02
	<2.2	32%	75%	
I-FABP	≧6.5	71%	6%	<0.001
	<6.5	29%	94%	

WBC: white blood cell; Plt: platelet; ALP: alkaline phosphatase; LDH: lactic dehydrogenase; CK: creatine phosphokinase; CRP: C-reactive protein; LA: lactic acid; I-FABP: intestinal fatty acid-binding protein.

### Multivariable logistic regression analysis

A multivariable logistic regression analysis into which the blood markers with a *P* value of less than 0.05 in the univariate analysis were entered (including the levels of WBC, LA and I-FABP) indicated an I-FABP level higher than 6.5 ng/ml to be the only independent significant factor for a higher likelihood of a diagnosis of strangulated SBO (P =  0.02; odds ratio: 19.826; 95% confidence interval: 2.1560 – 488.300) (Table 5).

**Table 5:** Results of the multiple logistic regression analysis.

	Cut-off value	Odds ratio	95% C.I	p-value
WBC	13700/μl	1.505	0.162–12.395	0.70
				
LA	2.2 mmol/l	1.938	0.202–15.867	0.54
				
I-FABP	6.5 ng/ml	19.826	2.1560–488.300	0.02
				

WBC: white blood cell; LA: lactic acid; I-FABP: intestinal fatty acid-binding protein.

## Discussion

Strangulated SBO is a very serious condition that requires a prompt diagnosis. Therefore, many previous studies have evaluated the early and accurate diagnosis of strangulated SBO, focusing especially on the differentiation of strangulated and simple SBO based on clinical signs, laboratory markers and CT findings or a combination of these parameters [2], [5]–[9], [26], [27]. Nevertheless, early detection remains difficult; therefore, the identification of more reliable diagnostic tools is required. I-FABP have a relatively low molecular mass of 14–15 kDa, which makes up a significant proportion (2%) of cytosolic proteins in enterocytes located at the top of intestinal villi [15], [28], [29]. Intestinal ischemic disease associated with intestinal hypoperfusion results in the rapid loss of epithelial cell membrane integrity, which allows I-FABP to leak into the circulation, resulting in elevation of the serum I-FABP concentration [30]. Recent clinical reports have demonstrated the usefulness of measuring the I-FABP level in order to accurately diagnose intestinal ischemia, with a sensitivity of 68–79% and a specificity of 71–74% [19], [21], [22], [31]. However, few studies have investigated the serum I-FABP concentrations in comparison between SBO patients with and without strangulation. Cronk et al. first evaluated the usefulness of I-FABP for detecting strangulated bowel obstruction [20]. That study showed that serum I-FABP was positive in all patients with small bowel necrosis requiring resection and in 22% of those without necrosis, when an I-FABP level of more than 100 pg/ml was defined as positive, with a sensitivity of 100%, specificity of 83%, PPV of 50% and NPV of 100% for the serum I-FABP level with respect to detecting strangulated mechanical SBO. The present study included more patients with strangulated SBO than previous studies and compared the usefulness of the serum I-FABP level with other biomedical markers, including the levels of LA, CPK and LDH, which were not included in the study by Cronk. Moreover, in our study, the area under the ROC curve for the I-FABP level was larger than that for LA, which has been reported to be a useful marker for predicting strangulated SBO (0.854 and 0.735, respectively) [32]. An increased level of serum I-FABP can be used to accurately and easily discriminate between cases of strangulation, which requires emergent laparotomy, including resection of the necrotic bowel, and simple SBO due to adhesion, most cases of which can be treated conservatively [33], [34].

Several experimental studies have demonstrated that the LA level is also a useful predictor of intestinal ischemia, and clinical reports have described this parameter to be a significant biomedical marker for the diagnosis of strangulated SBO [31], [32], [35]–[37]. In the present study, the LA levels were significantly higher in the patients with strangulated SBO than in those with simple SBO, although the LA level was not found to be an independent factor for discriminating between strangulated and simple SBO. LA is the metabolic end product of anaerobic glycolysis and is produced in all tissues; however, the locations with the greatest production are the skeletal muscles, brain, intestines and blood cells. The metabolism and clearance of LA occurs primarily via the liver and kidneys; therefore, dysfunction of these organs affects the LA level [38]–[40]. Moreover, most patients with SBO do not consume adequate meals, or even water, and suffer from frequent vomiting, thus tending to develop dehydration, which may result in cellular low perfusion and elevation of the LA level. Therefore, this parameter may be increased due to dehydration alone in patients with SBO, even those without strangulation. This phenomenon likely also applies to I-FABP; however, in the present study, elevation of the serum I-FABP level was found to be the only independent factor distinguishing strangulated from simple SBO. In addition, the probability of a time lag between the onset of intestinal ischemia and an increase in the LA level described in a report by DeLaurier should be noted [35].

Previous reports have described the usefulness of measuring the serum interleukin-6 (IL-6) level to detect strangulated SBO [36], [41]. Yamamoto et al. reported that, in their study, the plasma IL-6 levels were significantly higher in the patients who required bowel resection due to strangulation compared with those observed in the patients who underwent emergent laparotomy without resection and those managed successfully without surgery [36]. Moreover, the sensitivity and specificity of the IL-6 level in predicting strangulation in patients with SBO were 86% and 86%, respectively, and the authors concluded that a plasma IL-6 level higher than 40 pg/ml is a significant predictive factor. Firrozmand et al. also reported that patients with an ischemic bowel have significantly higher levels of IL-6 than those without bowel ischemia (146.6 vs. 45.9 pg/ml; P = 0.034). According to these reports, measuring the IL-6 level is likely useful for detecting strangulation, although some problems exist. First, an increase in the level of IL-6 produced by various cells, including T-cells, B-cells, macrophages, endothelial cells, fibroblasts and so on, is observed in a variety of chronic inflammatory diseases (i.e. rheumatoid arthritis, Castleman's disease, systemic lupus erythematosus, systemic sclerosis); therefore, an elevated IL-6 level is not specific to patients with bowel ischemia [42]. Second, the timing at which the concentration of IL-6 increases can vary. In a previous report by Yamamoto et al., seven (50%) of 14 patients with significantly higher levels of IL-6 who required emergent resection of the intestines underwent collection of blood samples more than 24 hours after admission [36]. In an experimental study by Akimoto et al., significant differences in the serum concentrations of IL-6 were observed between strangulated and non-strangulated obstruction models, although the difference did not appear until 12 hours into the experiment [43]. Based on these data, measuring the IL-6 levels is not appropriate for diagnosing strangulated SBO in the early phase. On the other hand, the expression of I-FABP is restricted to enterocytes of the small intestine and the level of I-FABP has been reported to dynamically reflect the state of the intestines. One experimental study in which an ischemic model was created by ligating the superior mesenteric artery (SMA) and performing 30 minutes of transient SMA occlusion showed that the serum I-FABP level increased explosively within one hour after ligation, thus reaching a peak two hours later and then gradually decreasing [17]. The authors emphasized that elevation of the serum I-FABP level was observed before the pathologic microscopic findings showed transmucosal necrosis. Another study found a correlation between splanchnic hypoperfusion induced by cycling exercise and the plasma I-FABP level, reporting that the I-FABP level increased rapidly 40 minutes after the initiation of cycling [44]. These studies suggest that the level of I-FABP reflects the dynamic state of the intestinal mucosa and can be used to easily and early discriminate between strangulated and non-strangulated SBO.

Procalcitonin (PCT), which is an accepted inflammatory biomarker, has also been reported to be a useful predictor of ischemia and/or necrosis in patients with acute bowel obstruction [45]. In that report, the PCT level exhibited a high positive predictive value of 95% and negative predictive value of 90% for intestinal necrosis; therefore, the authors concluded that measuring the PCT level is very useful for diagnosing or excluding strangulation. However, acute bowel obstruction is thought to cause bacterial translocation, even in cases of simple obstruction, by promoting bacterial overgrowth, increasing intestinal permeability and/or physically disrupting the mucosal barrier. Bacterial translocation increases the level of inflammatory mediators resulting in elevation of the PCT level; therefore, the potential for an elevated PCT level due to inflammation caused by bacterial translocation should be kept in mind, even in cases without strangulation [46], [47]. The present study did not perform the analysis including PCT levels because the measurement of PCT was not done for all of the patients, although further study including those measurements should be necessary to confirm the usefulness of I-FABP.

There are several limitations associated with this study. This study was a retrospective study with a small number of patients previously diagnosed with SBO. In addition, I-FABP occurs in the large bowel, although the amount is very small; therefore, measuring the I-FABP values, including those in patients with large bowel obstruction complicated with or without intestinal necrosis, is necessary to confirm that an increased I-FABP level is specific to strangulated SBO. Therefore, a prospective investigation with a large number of patients with acute abdomen in whom bowel dilatation is not clearly detected on imaging examinations is required to conclusively confirm whether the I-FABP level is a predictive laboratory marker of strangulated SBO. Such investigations will provide clinically important insight, including: 1) the ability to accurately diagnose strangulated SBO and provide prompt decision making with respect to the need for emergency surgery without confusion; 2) a reduced rate of unnecessary laparotomy leading to additional adhesion formation; and 3) an eventual reduction in both mortality and morbidity. Moreover, the measurement of the serum I-FABP level has not yet been introduced as a routine procedure in clinical practice. One reason for this phenomenon is that the present method for measuring this parameter using the ELISA system requires a long period of time. Therefore, such tests may sometimes not be appropriate, especially in cases in which emergent treatment is required. Further improvements in measuring methods are therefore required to shorten the time needed for measurement.

In conclusion, the present study demonstrated that the serum levels of I-FABP and LA are significantly higher in patients with strangulated SBO than in those with simple SBO. Furthermore, using the cut-off level calculated according to the ROC curve, an I-FABP level higher than 6.5 ng/ml was found to be an independent marker for discriminating between the two groups. Therefore, measuring the I-FABP level is useful for discriminating between strangulated and simple SBO.
